# A structural equation model linking health literacy, self efficacy and quality of life in adults with coronary heart disease

**DOI:** 10.1186/s12872-022-02720-8

**Published:** 2022-06-23

**Authors:** Shaoying Du, Zhimin Feng, Wen Wang, Licong Tian, Yan Wang

**Affiliations:** 1 School of Nursing, Hebei University, No. 342 Yuhuadong Road, Baoding City, 071002 Hebei Province People’s Republic of China; 2 School of Clinical Medicine, Hebei University, Baoding City, Hebei People’s Republic of China

**Keywords:** Coronary heart disease, Health literacy, Self efficacy, Quality of life, Structural equation modelling

## Abstract

**Background:**

Cardiovascular disease is the world major cause of death. There is sufficient evidence that patients with coronary heart disease (CHD) experience poor quality of life. Health literacy and self efficacy are modifiable psychosocial factors that could affect quality of life, and these factors should be considered as targets for intervention. As the relationships among health literacy, self efficacy, and quality of life in the CHD population have not been well understood. Thus, we constructed the structure equation model in these valuables.

**Methods:**

A cross-sectional study of a convenience sample among 200 patients with CHD were participated from outpatient clinics in three tertiary general hospitals in Baoding City in mainland China, from December 2018 to June 2019. Data regarding demographic features, health literacy, self efficacy and quality of life were assessed. A structure equation model was used to construct and validate the pathways.

**Results:**

The mean age of the study sampled patients was 65.37 years old. The average level of health literacy, self efficacy and quality of life were 9.6 ± 3.5, 28.8 ± 13.9 and 381.8 ± 130.1 respectively. Significant associations were observed from health literacy to quality of life, and self efficacy played a partial mediating role between health literacy and quality of life in the CHD population. Health literacy and self efficacy explained for 59.6% of the variance in quality of life.

**Conclusions:**

Health literacy had a direct influence on quality of life, and an indirect influence on quality of life via self efficacy in the patients with CHD.

**Supplementary Information:**

The online version contains supplementary material available at 10.1186/s12872-022-02720-8.

## Background

Cardiovascular disease (CVD) is the world 1^st^ leading cause of death in the twentieth century [1]. There are 290 million CVD patients in China, among which 11 million are coronary heart disease (CHD) patients [2]. Although the mortality have declined by the improved treatment, the CHD patients have to cope with the symptoms such as chest pain, breathing difficulties, as well as complex treatment options for a long time, which could negatively impact the patients’ satisfaction and physical and mental health. The dimensions of Quality of life (QOL) include physical and mental health. QOL is regarded as an important outcome of health care and is increasingly viewed as a comprehensive indicator of health in population health surveys and nursing interventions [3].

There is sufficient evidence that patients with CHD experience poor QOL comparing with the healthy population [4]. One research indicates that there is limited understanding of QOL and its influencing factors in CHD population in Asia [5]. Understanding the influencing factors of QOL is essential to alleviate the effect of CHD on public health. It was shown that QOL could be affected by many factors, such as socio-demographic factors (i.e., age, gender, education level and social support), physical factors (i.e., health status and comorbidity), and psychological factors (i.e., stress, anxiety and depression) [6–9]. However, some of these factors are objective, and they cannot be modified [8, 9]. Moreover, health care givers maybe limited from enhancing QOL, as only a partial picture of QOL and associated factors were given [6]. Therefore, to recognize the importance of an individual’s holistic perspective is needed.

HL is regarded as individuals’ capacity to obtain, process, understand the health information and make appropriate healthcare decisions [10]. Therefore, HL is an important part of secondary prevention in CHD patients. Because these patients could read and understand health education materials, adhere to medication regimes, make lifestyle changes, and manage their condition effectively by HL [11], which lead them to improving the QOL. Recent report has shown that HL is now emerging as a significant determinant to improve self-rated health [12].

HL was directly associated with QOL in research probing the association in patients with ischaemic heart disease [13] and in percutaneous coronary intervention (PCI) patients at 6 months after discharge [14]. However, the findings were inconstantly observed in different studies. One study in hospitalized CHD patients found that there was no direct correlation between HL and QOL, but only indirect correlation through the medicator of SE [15]. The mixed findings showed that the relationship among HL, SE and QOL might be complicated and require further clarification.

SE refers to an individual’s confidence in one’s ability to complete tasks [16]. Paasche-Orlow and Wolf proposed the framework of HL and health outcomes, and hypothesized that SE might link HL and QOL [17]. Empirical studies have found that HL is associated with QOL via SE in patients with chronic disease, such as hypertension [18] or diabetes mellitus [19]. Up to now, much research has been done on the relationship between QOL and its related variables through correlation or regression method, which could only reflect the direct effect on QOL, and little work has revealed the pathway between HL, SE and QOL in CHD patients. Therefore, the present research was to investigate the pathway among HL, SE and QOL, and to investigate direct and indirect effects among variables in CHD patients. Significant associations were hypothesized as follows: (1) HL was associated with QOL directly in CHD patients; (2) SE was the mediator between HL and QOL in CHD patients. The study may be used as the basis for developing interventions for improving the QOL in CHD patients.

## Methods

### Research designs and setting

A cross-sectional design was employed in the study. Patients who visited the outpatient clinic for post-discharge follow-up were selected through a convenience sampling following the STROBE guidelines from three tertiary general hospitals (the affiliated hospital of Hebei University, the first central hospital of Baoding, and the second hospital of Baoding) in Baoding City, Hebei province, mainland China. These hospitals are public, not referral, and each with > 500 beds. Meanwhile, they are teaching hospitals of Hebei University. The research was approved by the Institutional Review Board of Hebei University. All methods were performed in accordance with the Declaration of Helsinki.

### Participants

Participants who were selected for the study met the following criteria: (1) at least 18 years old; (2) diagnosis with CHD by a cardiologist (i.e., patients with myocardial infarction and/or PCI, angina) for more than 6 months; (3) able to give written informed consent. Participants who couldn’t communicate independently because of cognitive or mental impairement or had severe complications (i.e., class IV heart failure, renal failure, diabetes, malignant tumor) were excluded. Each patient could join the study only once.

The sample size formula is N = (u_α/2_ × σ/δ)^2^. We expected to observe HL level. After the preliminary experiment showed that the standard deviation of HL was 4.1, based on the power of 80% and α of 5%, therefore, the sample size was 101 [(1.96*4.1/0.8)^2^ = 101]. After 10% dropout rate was considered, the minimum estimation of final sample size was 111. Meanwhile, the sample included in structural equation modelling is minimum of 10 per indicator. The number of parameters to be esimated in this study was 17. As such, the minimum sample size is 170. A convinent sample of 200 patients could meet the sample size.

### Data collection and measures

The data were collected from December 2018 to June 2019. Patients who met the criteria and visited the outpatient clinic were viewed as potential participants. They were informed the study and invited to participate by the researchers. If they agreed, they would be required to sign a consent. After informed consent was obtained, each participant was given the questionnaire. Patients could finish the questionnaire independently or with the researchers’ help in 20–30 min in a quiet room in the outpatient clinic.

#### Socio-demographic data

Gender, age, marital status, education attainment, monthly household income, employment status, family history of CHD and PCI treatment were included.

#### Health literacy scale

HL was assessed using self-reported Brief Screening Questions, which was used in patients with CVD and other diseases [14, 20–22]. There were 3 items as follows: “how confident are you filling out medical forms?”; “how often do you have problems learning about your medical condition because of difficulty understanding writing information?”; and “how often do you have someone help you read hospital materials?”. Answers were based on a 5-point Likert scale (1–5). The scores ranged from 3 to 15, with lower scores reflecting higher HL. Cronbach’s α was 0.88 in this study indicating excellent internal consistency.

#### Self efficacy scale

SE was assessed using a scale for chronic disease, which was used in some studies [23]. It included 2 dimensions and 6 items. The scores ranged from 6 to 60, and the higher the score, the higher the subjects' SE. Cronbach’s α was 0.94 in this study indicating excellent internal consistency.

#### Health-related quality of life

The Chinese version of the 36-item Short Form was used to assess the QOL [24]. It included 8 dimensions, and could be viewed as 2 summaries: physical health and mental health. The raw scores of each dimension were converted to a range 0 to 100. The higher score gets, the better level of QOL is. Cronbach’s α was 0.89 in our study indicating excellent internal consistency.

### Ethical consideration

The research was approved by the Institutional Review Board of Hebei University, China. All participants could refuse or withdraw from the research at any time with no penalty.

### Data analysis

All data were analysed using SPSS, version 22.0 and AMOS, version 24.0. Types and prevalence of demographic data were described with frequency and percentages. Total HL, SE and QOL scores were described by means and standard deviation. The questionnaire would be excluded for absence of more than 3 data. The independent sample *t* test and ANOVA test were applied to analyze the difference in demographic characteristics of HL, SE and QOL. Pearson correlation was used to analyze the correlation between HL, SE and QOL. *P* values less than 0.05 indicated a significant difference.

The variables of HL, SE, and QOL were considered as latent constructs. The corresponding variables were considered as observed variables for each latent constructs. Since HL was measured in reverse, our assumptions were as follows: significant negative associations were observed from HL to QOL, from HL to SE, and possible associations were observed from SE to QOL. A structure equation model (SEM) was used to construct and validate the pathways. Comparative fit index (CFI) value above 0.9, root mean square error of approximation (RMSEA) value below 0.07, goodness-of-fit index (GFI) value above 0.9, adjust goodness-of-fit index (AGFI) value above 0.9, and the value of Chi-square/degrees of freedom (x^2^/df) below 3 indicated model fit.

## Results

### Distribution of related variables and associations among them

**Fig. 1:**
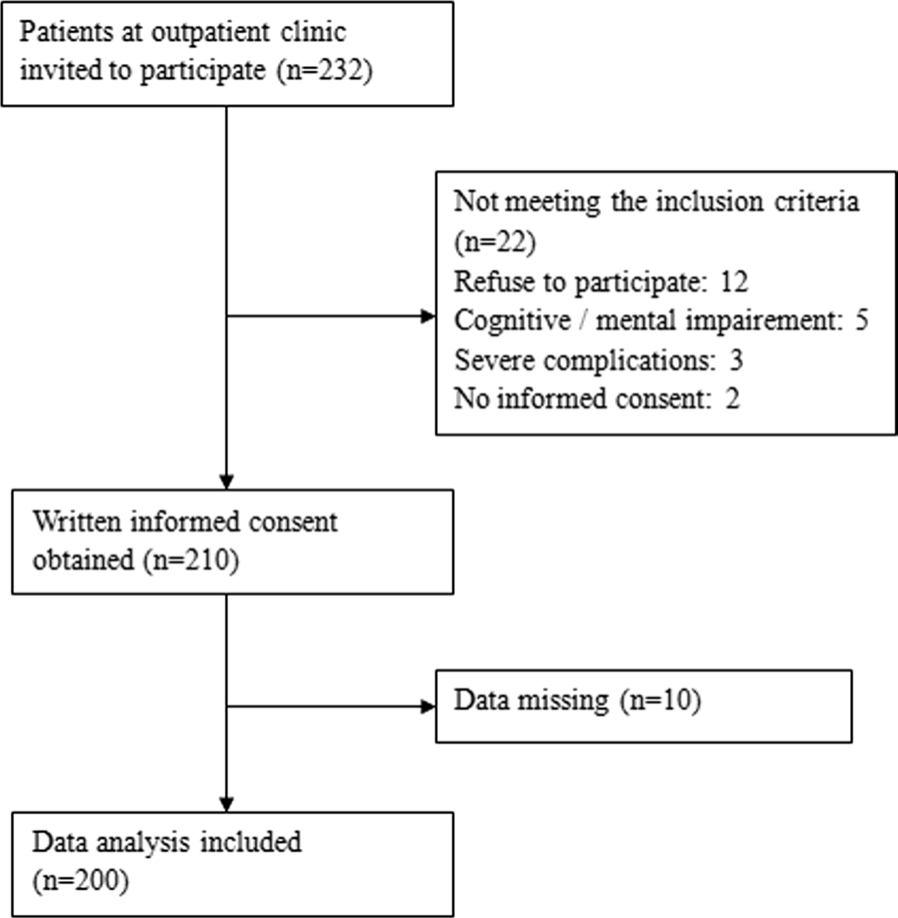
Sampling frame

Of 232 patients invited, 22 patients were excluded for not meeting the inclusion criteria. 10 patients were excluded for missing data. Ultimately, 200 patients completed the questionnaires (Fig. 1). Table 1 shows the distribution of personal characteristics and their associations with the research valuables. We found that there were significant differences in HL and QOL among people of different ages and genders. Also, patients with different levels of education, employment status and monthly incomes had different HL, SE and QOL (see details in Table 1). Table 2 shows the associations among HL, SE and QOL. Significant associations were also found between them (see details in Table 2).

**Table 1:** Personal characteristics and their associations with health literacy, self efficacy, and quality of life (n = 200)

Personal characteristics	Mean (SD) /n (%)	Health literacy Mean (SD)	Self efficacy Mean (SD)	Quality of life Mean (SD)
Age	65.51 (10.37)	9.6 (3.5)	28.8 (13.9)	381.8 (130.1)
*r* value		0.379**	− 0.121	− 0.187**
**Gender**				
Male	111 (55.5%)	8.2 (3.1)	30.1 (14.5)	402.9 (122.9)
Female	89 (44.5%)	11.3 (3.2)	27.2 (12.9)	355.7 (134.7)
*t* value		− 6.9***	1.4	2.6*
**Marital status**				
Single(includes divorced, widowed, separated)	13 (6.5%)	10.7 (3.4)	31.1 (13.7)	382.0 (108.3)
Married	187 (93.5%)	9.5 (3.5)	28.7 (13.9)	381.8 (131.7)
*t* value		1.2	0.6	0.005
**Education attainment**				
Less than junior high school	166 (83.0%)	10.3 (3.2)	27.6 (13.4)	367.0 (123.8)
Senior high school/college/equivalent	34 (17.0%)	6.2 (2.7)	34.6 (14.7)	454.2 (137.5)
*t* value		6.9***	− 2.7**	− 3.7***
**Income status (RMB/month)**				
Below 2000	65 (32.5%)	11.1 (3.0)	27.1 (12.3)	337.2 (116.7)
2000–3000	81 (40.5%)	9.5 (3.1)	28.3 (13.0)	381.8 (116.1)
Above 3000	54 (27.0%)	8.0 (3.9)	31.7 (16.4)	435.6 (146.2)
*F* value		12.23***	1.8	9.1***
**Employment status**				
Unemployed	81 (40.5%)	10.8 (3.3)	25.8 (12.5)	358.9 (122.0)
Retired	92 (46.0%)	9.6 (3.2)	29.6 (13.3)	373.4 (121.4)
Employed	27 (13.5%)	6.1 (2.7)	35.2 (17.0)	479.4 (142.8)
*F* value		22.9***	5.2**	9.9***
**Family history of CHD**				
Yes	62 (31.0%)	9.4 (3.8)	29.7 (15.1)	391.8 (141.9)
No	138 (69.0%)	9.7 (3.3)	28.4 (13.3)	377.4 (124.7)
*t* value		− 0.5	0.6	0.7
**PCI treatment**				
Yes	99 (49.5%)	9.5 (3.5)	29.8 (12.8)	389.4 (119.7)
No	101 (50.5%)	9.7 (3.5)	27.9 (14.9)	374.5 (139.7)
*t* value		− 0.4	0.9	0.8

*p < 0.05

**p < 0.01

***p < 0.001

**Table 2:** Correlation among health literacy, self efficacy, and quality of life (n = 200)

	Health literacy	Self efficacy	Quality of life
Health literacy	1.000	− 0.322***	− 0.481***
Self efficacy		1.000	0.734***
Quality of life			1.000

***p < 0.001

### Test of the model

**Fig. 2:**
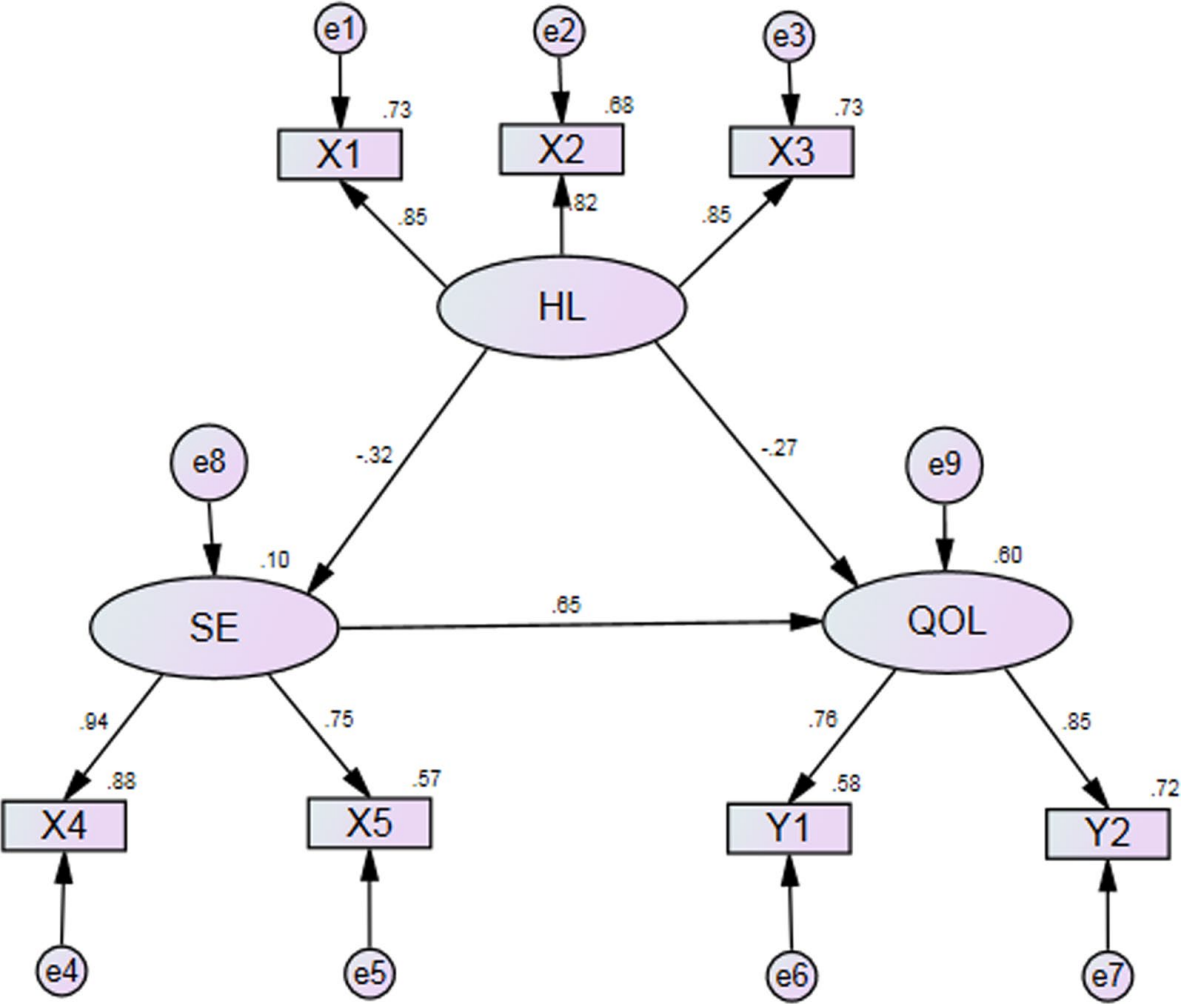
Final model and standardized pathway coefficients among health literacy, self efficacy, and quality of life. HL = health literacy; SE = self efficacy; QOL = quality of life; X1 = help read; X2 = confidence; X3 = problem learning; X4 = symptom management self efficacy; X5 = disease generic management self efficacy; Y1 = physical health; Y2 = mental health

The significant direct pathways were found from HL to QOL, and SE played a partial mediating role between HL and QOL in the CHD population (see details in Fig. 2).

The fit indices were x^2^/df = 0.966, GFI = 0.986, AGFI = 0.963, CFI = 1.000, and RMSEA = 0.000 [90% confidence interval (0.000–0.072)]. It indicated that the proposed model was acceptable. HL significantly indirectly affected QOL through SE. The direct effect of HL on SE was − 0.32, the direct effect of SE on QOL was 0.65, the direct effect of HL on QOL was − 0.27, the indirect effect of HL on QOL was − 0.21 (− 0.32*0.65), and the total effect of HL on QOL was − 0.48 (− 0.27–0.21). Therefore, the total effect was greater than the direct effect, the direct effect was greater than the indirect effect of HL on QOL, and SE only played a partial mediating role. Lastly, HL accounted for 10.4% of the total variance in SE. HL and SE explained for 59.6% of the total variance in QOL.

## Discussion

Several studies explored the relationship among HL, SE and QOL in patients with chronic disease [15, 25, 26]. However, correlation or regression method were often used, which could only reflect the direct effect on QOL. Up to now, little work has revealed the indirect effect among HL, SE and QOL in CHD patients. Therefore, current study constructed and validated the pathways between HL, SE and QOL in CHD patients by SEM.

HL was significantly correlated with QOL in CHD patients in this study, which is in line with previous findings [13, 14], suggesting that CHD patients with inadequate HL tend to have poor QOL. Patients with chronic diseases often require medication therapy and changes in lifestyle for a long time [27]. Inadequate HL was ralated with less understanding of medical conditions [28]. Therefore, inadequate HL may affect health status. The results reported by Son et al. [14] revealed that adequate HL is a contributing factor in improving QOL in patients after PCI.

SE was the mediator between HL and QOL in CHD patients. SE directly positively influenced QOL in CHD patients. The finding was consistent with previous findings [29, 30], suggesting that CHD patients with low SE tend to have poor QOL. Patients with higher SE may put in more effort, and have a great tendency in persisting in their attempts [31]. Also, our study confirmed that SE was the mediator between HL and QOL, revealing that CHD patients with inadequate HL have a lower SE in daily life, and ultimately leading to poor QOL, which echoes the previous framework proposed by Paasche-Orlow et al. [17].

Up to now, literature on HL in CHD patients is limited and the level of HL in these patients is generally low, accompanied by low QOL [32]. This study provides a model for describing the relationship among HL, SE and QOL in CHD patients. Health care givers can design intervention programs for CHD patients according to this model.

There are several limitations in the study. First, the pathways among HL, SE and QOL were from a cross-sectional study database. Accordingly, causal pathways in the model need to be confirmed through longitudinal and experimental studies. Second, a convenience sampling was used. Therefore, these findings cannot be extrapolated to CHD patients in other countries. Third, there are other modifiable factors that may influence QOL, such as depression, anxiety and social support [33], body mass index [34] and waist circumference [35]. Future research could expand the previous framework with the consideration of these variables. Fourth, the HL measurement in this study was functional, which didn’t include communicable and critical literacy, thus, may not be perfect. Nevertheless, functional HL is the most commonly used measurement in mainland China [36]. In future studies, these factors should be considered.

## Conclusions

In this investigation, the aim was to assess the relationship among HL, SE and QOL in CHD patients, and to investigate direct and indirect effects among variables. SE directly positively influenced QOL values and HL and SE accounted for 59.6% of the total variance. HL not only directly affects QOL, but also indirectly affects QOL via SE. Health care givers can manage CHD patients based on the findings of our study. Because SE directly influences QOL, modifying SE is essential for improving QOL in CHD patients. To improve SE, a potential strategy is to enhance health literacy.

## Supplementary Information

**Additional file 1:** Raw data SPSS.

**Additional file 2:** Raw data Excel.

## Data Availability

All data generated or analysed during this study are included in this published article [and its Additional files 1 and 2].

## Abbreviations

AGFI: Adjust goodness-of-fit index
CFI: Comparative fit index
CHD: Coronary heart disease
CVD: Cardiovascular disease
GFI: Goodness-of-fit index
HL: Health literacy
PCI: Percutaneous coronary intervention
QOL: Quality of life
RMSEA: Root mean square error of approximation
SE: Self efficacy
SEM: Structure equation model
x^2^/df: Chi-square/degrees of freedom
